# Bones and Backbones

**Published:** 1888-05-05

**Authors:** 


*(To be continued.)*

### Physiology and its Uses.

### BONES AND BACKBONES.

A sculptor carves the image of a man out of a single piece of marble: nature uses at least two hundred separate pieces of bone to hang a single man's muscles upon, and to encase certain parts which, like glass and pictures, require to be handled "with care." Bones are not held of much account either by the grave-digger or the cook; but that is because the grave-digger and the cook are both very unscientific persons. There is a story, familiar to all schoolboys, to the effect that a countryman, visiting the museum of his county town for the first time, was shown the skeleton of an ass. He gazed with open-eyed wonder at the many separate pieces, the peculiar joints, and the various cavities made by the articulated bones; finally expressing his admiration in the scientific language most familiar to himself: "Ah, sirs, we are fearfully and wonderfully made." The laugh is held to have been against the countryman, because he placed the ass in the same order as himself. He was, however, a genuine Darwinian before Darwin's time.

The higher monkeys have bones very similar to our own, and in many respects identical with them. Not only so, but all backboned animals have them too. But, says an objector, monkeys, donkeys, and most other backboned creatures have tails—Where are our tails? The tail argument is long since exploded. At a certain stage of development there is in every normal human being a projection of the last bones of the vertebral column exactly "like a true tail," as Darwin says, on the authority of Professor Wyman, which tail extends "considerably beyond the rudimentary legs." Nay, more—and this finishes the discussion—"All the bones in a man's skeleton can be compared with corresponding bones in a monkey, bat, or seal."

It is worth while bearing in mind that a large portion of the animal kingdom is destitute of bones. Who has not eaten a whole oyster at a gulp? Where were his bones, where his brain, his stomach, and his liver? He had no bones. True, he had an outer protecting case—a skeleton of a kind; but no bones in his own proper structure. And it is quite possible to imagine a perfect human being without bones: that is, a human being possessed of mind and morals of the same order as he is now distinguished by. Why not? The bones do not think or reason; they simply constitute a framework.

But though a man might easily think and have a conscience without bones, it is difficult to see how he could live in the same environment and play quite the same part in the world as he does now. Every creature known to us is more or less suitably adapted to his own peculiar environment; and man, playing undoubtedly the highest and most important part of all the animals, is adapted in a pre-eminent degree to his. This is easily demonstrated by the one single fact that man can survive under a greater variety of circumstances than any other living creature.

The basis of the human framework, as of all other vertebrate animal frameworks, is the backbone—the vertebral column. This is, so to speak, the foundation-stone on which all the rest of the building is raised; or it might be called, with almost equal propriety, the seed from which all the other parts of the bony tree grow. It is the first to appear as we ascend from the backboneless animals to the backboned; and it is the first to begin in each individual body. In an adult man there are 26 pieces in the vertebral column; in the child before birth there are 33. None of these 33 disappear as development proceeds, but the four lowest are fused together to form the *coccygeal bone*, and the next five to form what is called the *sacrum*. The remaining 24 are free and separate, except that they are united by soft structures called ligaments.

The backbone is a pillar and a box. As a pillar it supports the head, and has attached to it the shoulders and ribs. As a box it contains the spinal cord, or marrow. It is decidedly a wonderful box; for although it protects most strictly the spinal marrow, an injury to which may cause a great variety of mischiefs, up to complete paralysis of the lower extremities, yet it is capable of all sorts of wriggling and twisting movements without risk to its valuable contents. The wrestler, the acrobat, and the mischievous schoolboy test both its wriggling and protecting capacity to the utmost, and it very seldom fails to do all that is required of it.

This pillar-box deserves much more consideration at the hands of its owners than it usually receives. The general public seldom hear of diseases of the backbone, yet diseases and injuries here may be very serious and dangerous. Not long ago a wealthy woman of forty-eight died solely as the result of a long-standing curve in the spine. If she had been carefully watched by her parents when a child, and properly treated as soon as the curve began to make its appearance, she might have been alive and well at the present time. This kind of case is eminently one in which a little medical knowledge would be of the greatest possible value to both parent and child; and any one who would grudge such knowledge to intelligent general readers is a disgrace both to medicine and morals. Growing children, girls and boys, often complain of being tired, want to lie down, are irritable, and refuse their usual food. There does not appear to be much the matter with them, and unintelligent parents think they are lazy or "naughty." They should be taken to a competent medical man, stripped, and thoroughly examined. In these days spinal instruments are made very light and comfortable, and such an instrument will often restore the peevish child to perfect health in a few months.

The erect posture is natural to man, and it is to him much the most healthy posture. The backbone has no power of keeping itself erect, and yet it is very desirable that it should be kept so. The head, placed on the top of the vertebral column, is a heavy body, and is easily moved out of the perpendicular. The backbone, being in a position of very unstable equilibrium, is deflected on a small provocation, and unless a good deal of pains is taken, soon contracts a permanent stoop. A stoop is unsightly; but it is also injurious. It tends to contract the chest and to displace its contents—the heart and lungs. Such displacement may be highly mischievous. The backbone, however, may be made to keep its place without much difficulty. A proper amount of exercise for the arms and back are all that are required in the healthy child. The habits of the children of prosperous parents are not always conducive to straight backs. Boys brought up in the country and early put to farm labour, or to mechanical trades, seldom suffer from spinal complaints. Their work is their salvation. Children, both boys and girls, who are not required to work, should have intelligent gymnastic exercises taught them, and should be made to practise them daily. The recent testimony of famous acrobats of both sexes goes to show that abundance of vigorous and varied exercise not only strengthens the muscles of the back and keeps the backbone erect and supple, but also develops and invigorates the physical powers generally.

The average length of the human backbone is about twenty-eight inches, but of course it varies considerably. The height of a man, however, generally depends on the length of the legs rather than on that of the body. There are quite little men who, if their legs were of the same relative length as their bodies, would be more than six feet high. We cannot, as yet, tell them of any safe process for elongating the legs.

It will be of interest to many readers to know that Goethe pursued the scientific study of human development with much energy and critical ability. He is believed to have been the first to originate the notion that the bones of the head are really portions of the backbone, or modified vertebræ, altered in the course of growth to serve their special and peculiar purpose. Regarding this question much has been said on both sides, but scientific anatomists are largely agreed that Goethe's critical intellect served him well. This is not the place for offering reasons for or against theories which belong of right to the laboratory and the study; but it is well to bear in mind that no fact of science is isolated or insignificant, but that each is related to the whole; and it is only by an intelligent insight into the general bearing of all classes of facts that we can take a comprehensive and intelligent view of life and the world as they really are.

